# Complex partial non-convulsive status epilepticus masquerading as hepatic encephalopathy: a case report

**DOI:** 10.1186/1752-1947-6-422

**Published:** 2012-12-17

**Authors:** Maaz B Badshah, Haris Riaz, Sana Aslam, Moaviz B Badshah, Mark A Korsten, Muhammad Bilal Munir

**Affiliations:** 1 James J. Peters VA Medical Center/Mount Sinai School of Medicine, New York, NY, USA; 2 Civil Hospital Karachi, Karachi, Pakistan; 3 Khyber Medical University, Peshawar, Pakistan; 4 Department of Medicine, University of Pittsburgh Medical Center, Pittsburgh, PA, USA

**Keywords:** Hepatic encephalopathy, Non-convulsive status epilepticus, Partial seizures, Electroencephalogram

## Abstract

**Introduction:**

Hepatic encephalopathy is usually suspected in patients who are cirrhotic with neuropsychiatric manifestations. We present a case of suspected hepatic encephalopathy that did not respond to standard empiric therapy and was eventually diagnosed as non-convulsive status epilepticus of complex partial type. Our patient responded dramatically to anti-convulsive therapy.

**Case presentation:**

We report the case of a 45-year-old African-American man with hepatitis C virus cirrhosis and human immunodeficiency virus who presented to our facility with a one-day history of confusion and a variable mental status. Our patient’s vital signs were stable and all his electrolytes were within normal range. A clinical diagnosis of hepatic encephalopathy was made and our patient was started on empiric therapy with lactulose and rifaximin. Our patient did not respond to therapy. After five days of treatment, alternative diagnoses were sought and a neurology consult was requested. An electroencephalogram was eventually performed which showed seizure activity in the right parietal lobe. A diagnosis of non-convulsive status epilepticus was made and our patient was started on oral levetiracetam. On day two of therapy, our patient was alert and oriented. He continues to do well on follow-up approximately one year after discharge.

**Conclusions:**

Non-convulsive status epilepticus should be considered in the differential diagnosis of patients with suspected hepatic encephalopathy who do not respond to empirical treatment. Further studies are needed to investigate the incidence of this entity in patients with persistent hepatic encephalopathy.

## Introduction

Hepatic encephalopathy (HE) is a well known clinical entity and should be suspected in any patient with cirrhosis having neuropsychiatric manifestations in the absence of a brain lesion [1].

Its management depends on the prompt recognition of the precipitating factors and initiation of the empirical therapy.

Non-convulsive (or subclinical) seizures are not accompanied by any sensory or motor phenomenon but can be recognized as discharges in the electroencephalogram (EEG) [2]. Due to the lack of overt seizure activity, the condition tends to be under-diagnosed. In the present work, we report the case of a patient with a high clinical suspicion of hepatic encephalopathy who was eventually diagnosed as having non-convulsive seizures. To the best of our knowledge, this is the first reported case of such a presentation.

## Case presentation

A 45-year-old African-American man presented to our Emergency Department in a delirious state with loss of memory for one day. He had a past medical history of human immunodeficiency virus (HIV) (on highly active antiretroviral therapy (HAART), compliant, since 1999), hepatitis C virus (+cirrhosis, -Epstein-Barr virus, -Spontaneous bacterial peritonitis, since 2002) and hypertension for 10 years. Two weeks prior to presentation, he had an episode of hepatic encephalopathy for which he was treated with rifaximin and lactulose and was subsequently discharged on these medications clinically improved. Upon arrival, our patient was confused and his mental status was waxing and waning. He was able to tell us his name, date of birth and address but was unable to reveal how he was feeling. Our patient was drowsy and had to be woken up several times to answer questions. His blood pressure was 130/70mmHg, pulse 56 beats/min, and oxygen saturation 99 percent.

Our patient could not recall what drugs he was taking. He used to smoke four cigarettes per day and there was no history of alcoholism.

On examination, he looked lethargic, his face and extremities were cold, his neck was supple and spider nevi were seen on the left side of his neck and right shoulder. A chest examination was clear to auscultation and percussion. His heart sounds were normal. He was alert and oriented but his mental status was variable. Sensation was intact bilaterally, and there was no asterixis. There was sloughing of the skin over the left leg due to chronic venous changes; scaling was present on the plantar surfaces of both feet, and the dorsalis pedis pulses were intact bilaterally.

Laboratory investigation results showed hemoglobin 10.4g/dL, hematocrit 31.4 percent, white blood cells 7700 cells/mm^3^, platelets 135,000 cells/mm^3^; Na+ 130mEq/L, K+ 4.9mEq/L, Cl- 103mEq/L, CO_2_ 24.0mmHg, albumin 1.9g/dL, bilirubin 2.6mg/dL, alkaline phosphatase 179IU/mL, alanine transaminase 221IU/mL, aspartate transaminase 87IU/mL, corrected anion gap, 8.7, international normalized ratio 1.96, activated partial thromboplastin time 41.1. The ammonia level was noted to be approximately 50. Results of a chest X-ray showed cardiomegaly.

**Figure 1:**
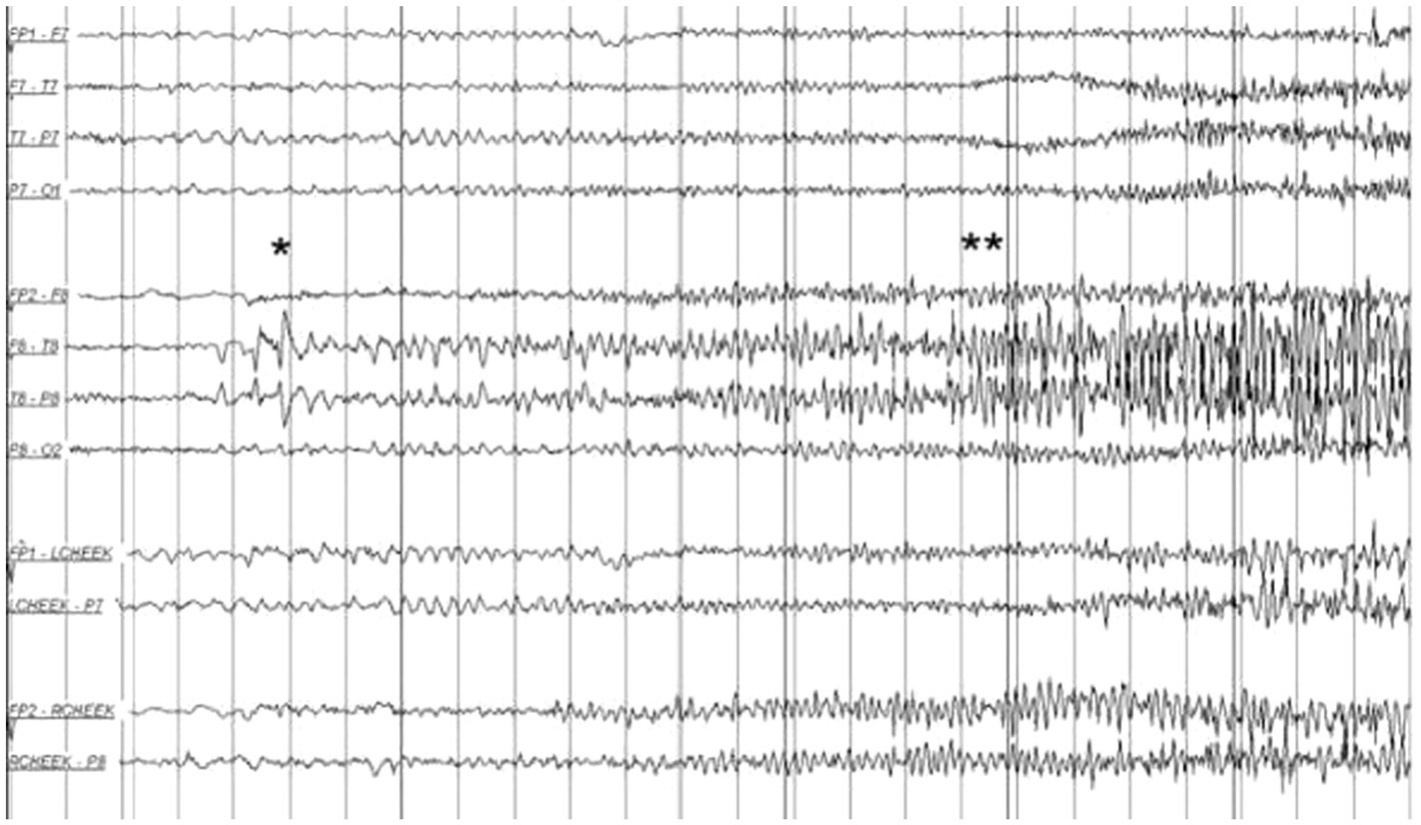
**Electroencephalogram showing seizure activity in the right parietal lobe.** The "*" denotes the onset and buildup and "**" denotes the progression of focal epileptiform discharges.

Our patient’s condition and laboratory test results suggested that his altered mental state was secondary to hepatic encephalopathy in the setting of cirrhosis secondary to chronic hepatitis C. He was given tablet rifaximin 400mg every eight hours and lactulose 30mg every six hours titrated to three to four bowel movements daily. A chest X-ray revealed only cardiomegaly, blood and urine culture results were negative, and the results of a metabolic profile were unremarkable. Our patient was initially started on empiric broad-spectrum antibiotic treatment with vancomycin 1g every 12 hours and piperacillin/tazobactam 3.375g every six hours, both of which were discontinued on day two of therapy when culture results were reported as negative. However, our patient’s mental status did not improve. On day six of hospitalization, a neurology opinion was sought. As part of the investigation, an EEG was performed which revealed ongoing seizure activity from the right parietal lobe (Figure 1).

A diagnosis of non-convulsive status epilepticus was made and our patient was started on oral levetiracetam 500mg every 12 hours. Within one day of initiation of this treatment, our patient became alert and oriented. According to our patient, he never had seizures in the past. A computed tomography (CT) scan of his head revealed an old left basal ganglia hemorrhage but no structural lesions in the right parietal lobe (from where the seizures seemed to originate). Our patient was subsequently discharged and continues to do well on follow-up one year after the discharge.

## Discussion

Hepatic encephalopathy is a common diagnosis when a patient with chronic active viral hepatitis presents with an altered level of consciousness. Symptoms of HE generally include disorientation, confusion and poor orientation. Although not totally defined, the pathophysiological basis of the condition is an interaction between elevated blood ammonia levels and astrocyte swelling [3].

Our patient was tentatively diagnosed as having HE after presenting with altered levels of consciousness. Empiric treatment with rifaximin and lactulose was thus initiated but the condition failed to improve. A subsequent EEG showed seizure activity in the right parietal lobe and a diagnosis of non-convulsive (subclinical) partial seizures involving the right parietal lobe was made.

Non-convulsive status epilepticus (NCSE) is defined as seizure patterns that are not accompanied by any alteration in the sensory phenomenon, motor functions or consciousness in the awake patient but are recognized as seizure activity in the electroencephalogram [4]. Owing to the diverse clinical presentation and the necessity of EEG for the diagnostic purpose, the condition has a tendency to be under-diagnosed.

The condition was defined by the Epilepsy Research Foundation Consensus Workshop in 2004 as ‘a term used to denote a range of conditions in which electrographic seizure activity is prolonged and results in non-convulsive clinical symptoms’ [5].

It is further categorized as absence, simple partial, and complex partial NCSE in patients who are comatose. While other neurological manifestations such as status epilepticus [6] and non-convulsive status epilepticus in patients who are comatose [7] masquerading HE have been rarely reported in the literature, the authors did not find complex partial non-convulsive seizures mimicking the condition in the medical literature.

The other reported case of NCSE was in a 64-year-old patient who was presumed to be suffering from HE secondary to chronic hepatitis C. This patient was ventilated mechanically and was sedated with midazolam while phenytoin infusion was used to treat the NCSE. Our patient did not require mechanical ventilation and the partial seizures responded well to levetiracetam. The presence of NCSE has been documented in several studies [8-10] to worsen the outcome since the processes resulting from NCSE can lead to or worsen the pre-existing brain injury. The case highlights the difficulty in diagnosing the condition and highlights the important role of EEG in aiding the diagnosis.

## Conclusions

Non-convulsive status epilepticus should be considered in the differential diagnosis of patients with suspected hepatic encephalopathy who do not respond to empirical treatment. Further studies are needed to investigate the incidence of this entity in patients with persistent hepatic encephalopathy.

## Consent

Written informed consent was obtained from the patient for publication of this case report and any accompanying images. A copy of the written consent is available for review by the Editor-in-Chief of this journal.

## Competing interests

The authors declare that they have no competing interests.

## Authors’ contributions

MBB analyzed and interpreted the data, HR, SA and MBB wrote the initial draft, MAK and MBM edited the manuscript. All the authors read and approved the final manuscript.
